# Supplementary material for: Smartphone videos of the sit-to-stand test predict osteoarthritis and health outcomes in a nationwide study
Source: NPJ Digit Med. 2023 Mar 4;6:32. doi: 10.1038/s41746-023-00775-1 (PMC9985590; doi:10.1038/s41746-023-00775-1)
Supplement: Supplementary file 1 — Supplementary Information [file 41746_2023_775_MOESM1_ESM.pdf]

Supplementary Information

Fig. Suppl. 1. Step-by-step details of the user-facing side of the web application. Note: The individuals in these photos gave written informed consent for the photos to be used in publication.

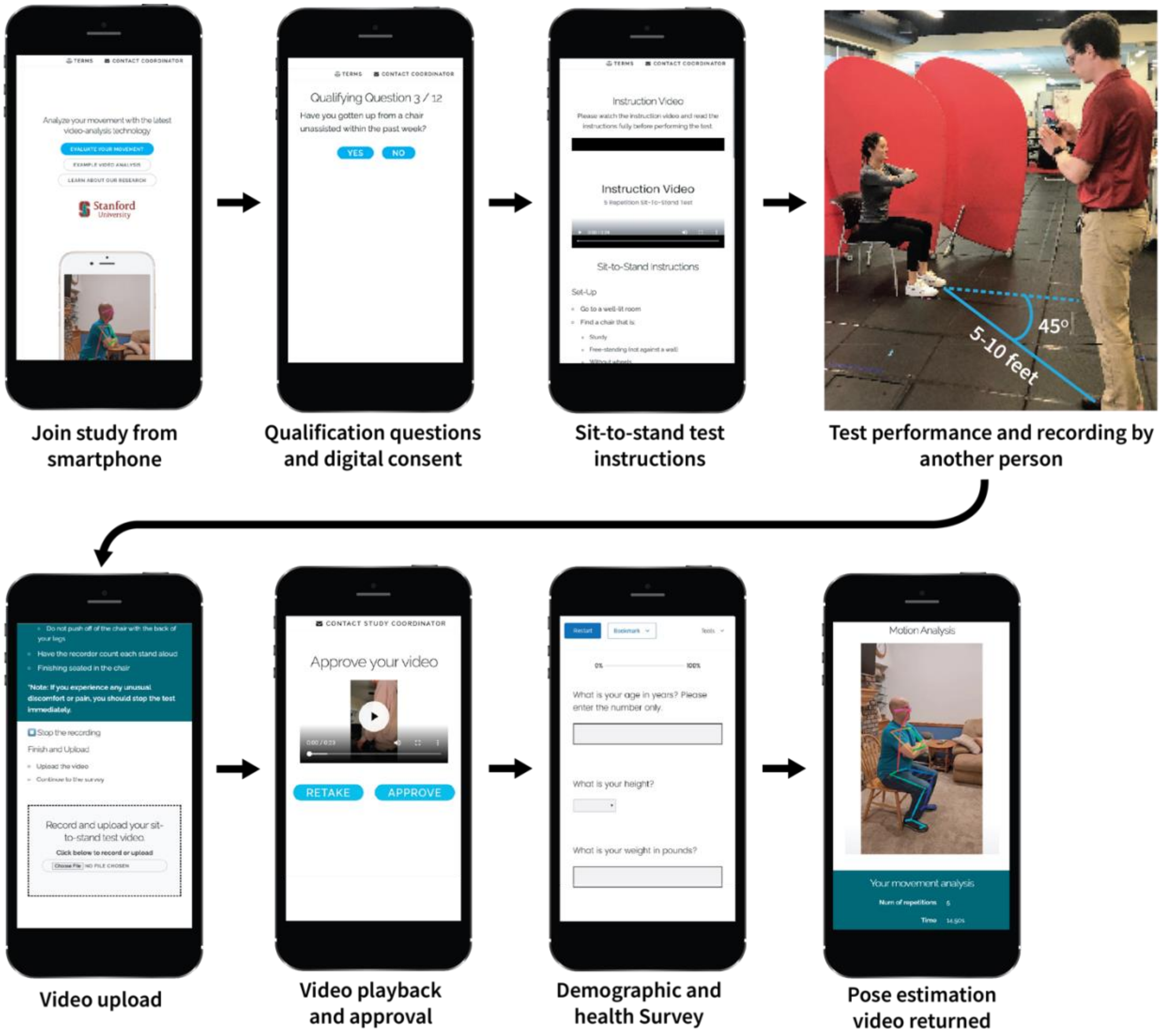

Fig. Suppl. 2. Flowchart of data included in the final analysis.

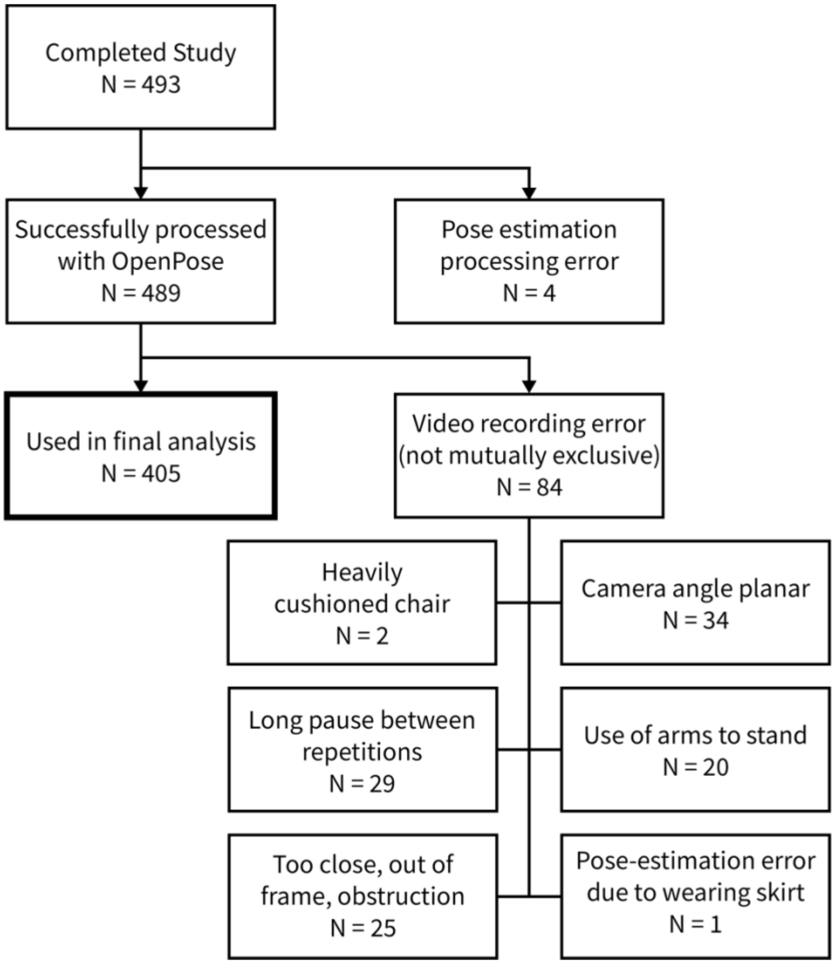

Fig. Suppl. 3. The five-repetition sit-to- stand test. Individuals start sitting down with their arms crossed in front of their chest and their feet flat on the floor. They then rise to stand (sit-to-stand transition) and sit back down (stand-to-sit transition) five times as quickly as possible.

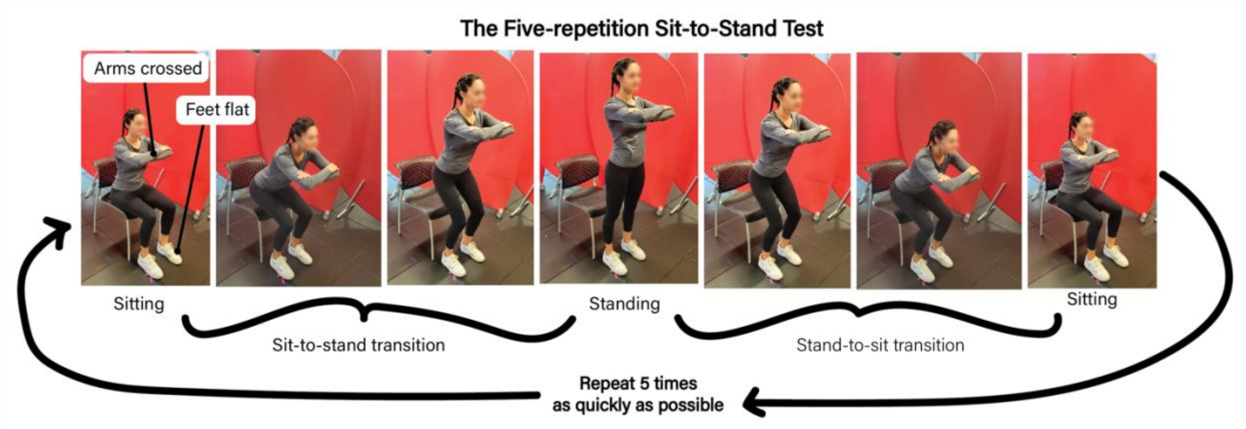

Table Suppl. 1. Participant characteristics.

Variable	18+ Years N = 405	50+ Years N = 106
**Participant Characteristics**		
Age (years), mean ± SD (min, max)	37 ± 18 (18, 96)	64 ± 10 (50, 96)
Age binned, n (%)*		
18-29 years	212 (52%)	-
30-39 years	62 (15%)	-
40-49 years	25 (6%)	-
50-59 years	44 (11%)	44 (42%)
60-69 years	35 (9%)	35 (33%)
70 + years	27 (7%)	27 (25%)
Sex, n (%)		
Male	191 (47%)	48 (55%)
Female	214 (53%)	58 (45%)
Gender, n (%)		
Male	192 (47%)	48 (55%)
Female	209 (52%)	58 (45%)
Non-Binary	2 (<1%)	0 (0%)
Other	1 (<1%)	0 (0%)
Prefer not to answer	1 (<1%)	0 (0%)
Height (in), mean ± SD (min, max)	67.3 ± 4.0 (55, 77)	66.5 ± 3.8 (55, 75)
Body weight (lbs), mean ± SD (min, max)	158 ± 34 (70, 280)	164 ± 34 (70, 280)
Ethnicity, n (%)		
White	243 (60%)	79 (75%)
Black or African American	14 (3%)	1 (1%)
Hispanic, Latino, or Spanish Origin	16 (4%)	2 (2%)
Asian	103 (25%)	19 (18%)
Mixed race	24 (6%)	2 (2%)

Other	5 (1%)	3 (3%)
Education, n (%)		
Less than high school	0 (0%)	0 (0%)
High school graduate	16 (4%)	4 (4%)
Some college	49 (12%)	11 (10%)
College Degree or Higher	338 (84%)	89 (84%)
Prefer not to answer	2 (<1%)	2 (2%)
Employment, n (%)		
Employed full time (40+ hours per week)	190 (47%)	46 (43%)
Employed part time (< 40 hours per week)	25 (6%)	9 (9%)
Student	129 (32%)	1 (1%)
Unemployed	9 (2%)	3 (3%)
Self-employed	13 (3%)	10 (9%)
Retired	33 (8%)	33 (31%)
Homemaker	3 (<1%)	2 (2%)
Prefer not to answer	3 (<1%)	2 (2%)
Income, n (%)		
< $10,000	17 (4%)	0 (0%)
$10,000 to $24,999	12 (3%)	0 (0%)
$25,000 to $49,999	53 (13%)	4 (4%)
$50,000 to $99,999	98 (24%)	25 (24%)
> $100,000	153 (38%)	48 (45%)
Prefer not to answer	72 (18%)	29 (27%)
State of residence (total #)	35	25
**Health Measures**		
BMI (kg/m^2), mean ± SD (min, max)	24.3 ± 4.1 (16.3, 41.0)	25.9 ± 4.5 (18.6, 40.2)
Physical health (T-score), mean ± SD (min, max)	54.9 ± 6.7 (32.4, 67.7)	53.3 ± 7.1 (32.4, 67.7)
Mental health (T-score),	53.0 ± 7.4 (28.4, 67.6)	54.6 ± 7.0 (38.8, 67.6)

mean ± SD (min, max)		
Osteoarthritis (Knee or Hip; Yes), n (%)	30 (7%)	29 (27%)
Hip	8 (2%)	8 (8%)
Knee	26 (6%)	25 (24%)
**5 STS Measures**		
Total time (s)	11.4 ± 3.4 (4.3, 32.9)	13.2 ± 4.3 (5.0, 32.9)
Maximum trunk angle during sit-to-stand transition (deg)	200.5 ± 8.3 (180.9, 239.3)	201.9 ± 9.9 (180.9, 239.3)
Maximum trunk angular acceleration during sit-to-stand transition (deg/s^2)	685.9 ± 162.7 (297.3, 1279.4)	678.7 ± 158.2 (297.3, 1216.4)

*Rounded percentages may result in sums slightly above or below 100%.
Abbreviations: BMI, Body mass index; Physical Health, PROMIS Global-10: Global Physical Health; Mental Health, PROMIS Global-10: Global Mental Health;

Table Suppl. 2. Pearson correlations between measures and five-repetition sit-to-stand test time and kinematics for all participants (N = 405). P-values account for multiple comparisons using a false discovery rate for 21 comparisons.

	**Time**	**Trunk Angle**	**Trunk Angular Acceleration**
**Age**	0.35****	0.15*	-0.02
**Sex**	0.03	0.00	0.05
**BMI**	0.20***	0.07	-0.02
**Ethnicity**	0.05	0.16**	0.01
**Physical Health**	-0.20****	-0.01	-0.04
**Mental Health**	0.02	0.06	-0.01
**Osteoarthritis**	0.18**	0.18**	0.06

* $P < 0.05$

** $P < 0.01$

*** $P < 0.001$

**** $P < 0.0001$

Abbreviations: BMI, Body mass index; Physical Health, PROMIS Global-10: Global Physical Health; Mental Health, PROMIS Global-10: Global Mental Health

Table Suppl. 3. Logistic regression analysis on a diagnosis of knee or hip osteoarthritis (N=405).

Dependent Variable	Independent Variables	β (SE)	OR	(95% CI)	P
Osteoarthritis (yes/no = 1/0)	Age	0.119 (0.012)	1.126	(0.094, 0.143)	<0.001
	Sex	-0.020 (0.023)	0.980	(-0.066, 0.026	0.393
	BMI	0.008 (0.012)	1.008	(-0.016, 0.031)	0.528
	Time	0.001 (0.012)	0.999	(-0.025, 0.024)	0.957
	Trunk Angle	0.029 (0.012)	1.030	(0.007, 0.052)	0.012

Abbreviations: BMI, Body mass index

Table Suppl. 4. Results of a Kruskal-Wallis rank sum test and post hoc Dunn's test for differences in trunk angle between races and ethnicities (N=405). P-values account for multiple comparisons using a false discovery rate for 6 comparisons.

Dunn's Test				Kruskal-Wallis Test		
**Comparison**	**Z**	**P**	**P_{adj}**	**χ^2**	**df**	**P**
Asian – Black	-3.653	<0.001	<0.001	25.47	3	<0.001
Asian – Hispanic	0.172	0.864	0.864			
Black – Hispanic	2.969	0.003	0.006			
Asian – White	-4.025	<0.001	<0.001			
Black – White	2.064	0.039	0.059			
Hispanic – White	-2.012	0.044	0.053			

Table Suppl. 5. Logistic regression analysis on race (N_{White}=243; N_{Asian}=103).

Dependent Variable	Independent Variables	B (SE)	OR	(95% CI)	P
Ethnicity (White = 0; Asian = 1)	Age	-0.043 (0.026)	0.958	(-0.094, 0.007)	0.093
	Sex	0.013 (0.047)	1.013	(-0.079, 0.105)	0.781
	BMI	-0.114 (0.024)	0.892	(-0.162, -0.066)	<0.001
	Time	-0.018 (0.025)	0.982	(-0.068, 0.031)	0.467
	Physical Health	-0.086 (0.024)	0.918	(-0.132, -0.039)	<0.001
	Trunk Angle	-0.084 (0.023)	0.920	(-0.130, -0.038)	<0.001

Abbreviations: BMI, Body mass index; Physical Health, PROMIS Global-10: Global Physical Health

Table Suppl. 6. Pearson correlations between measures and five-repetition sit-to-stand test time and kinematics for participants 50 years of age or older (N = 106). P-values account for multiple comparisons by controlling for the false discovery rate for 21 comparisons.

	**Time**	**Trunk Angle**	**Trunk Angular Acceleration**
**Physical Health**	-0.39***	-0.21	0.09
**Mental Health**	-0.06	0.09	0.28*

* *P* < 0.05

** *P* < 0.01

*** *P* < 0.001

**** *P* < 0.0001

Abbreviations: BMI, Body mass index; Physical Health, PROMIS Global-10: Global Physical Health; Mental Health, PROMIS Global-10: Global Mental Health

Table Suppl. 7. Linear regression analysis on mental health score for individuals 50 years of age or older (N=106).

**Dependent Variable**	**Independent Variables**	**β**	**(95% CI)**	**P**	**Adj. R^2**	**F (P)**
Mental Health (T-Score)	Age	0.803	(-0.632, 2.238)	0.270	0.108	3.544 (0.005)
	Sex	-1.874	(-4.492, 0.744)	0.159		
	BMI	-1.539	(-2.898, -0.180)	0.027		
	Time	-0.136	(-1.569, 1.297)	0.851		
	Trunk Angular Acceleration	1.705	(0.376, 3.034)	0.012		

Abbreviations: BMI, Body mass index

Doc. Suppl. 1. Sit2stand.ai Website Content

Home Page

[See page here]

Analyze your movement with the latest video-analysis technology

- JOIN OUR STUDY
- EXAMPLE VIDEO ANALYSIS
- LEARN ABOUT OUR RESEARCH

Want to be part of one of the largest human movement studies to date?

It's as simple as:

1) Recording a video with 5 sit-to-stand movements

2) Filling out a questionnaire

Participate in this Stanford University study to advance scientific research on health and well-being.

- JOIN OUR STUDY

Our research was supported by the Mobilize Center, a National Institutes of Health Big Data to Knowledge (BD2K) Center of Excellence through Grant U54EB020405, and RESTORE Center, a National Institutes of Health Center through Grant P2CHD10191301.

Doc. Suppl. 1. Sit2stand.ai Website Content

Before you start

Please confirm:

- You are using your mobile device (e.g., phone, tablet, iPad)
- You have 20 minutes to complete the entire study (*Note:* if you close this screen, you will have to start the study from the beginning)
- Your mobile device is equipped with a working video camera
- You have another person with you to record your test
- You have a sturdy chair with a back and without wheels

YES/NO

Doc. Suppl. 1. Sit2stand.ai Website Content

Qualifying Questions

Questions

1. Do you currently reside in the United States?
2. Are you over the age of 18?
3. Have you gotten up from a chair unassisted within the past week?
4. Do you feel safe sitting down on and standing up from a chair without the use of your arms?
5. Are you with another person who can monitor your participation and record your video?
6. Has your doctor ever said that you have a heart condition and that you should only do physical activity recommended by a doctor?
7. Do you feel pain in your chest when you do physical activity?
8. In the past month, have you had chest pain when you were not doing physical activity?
9. Do you lose your balance because of dizziness or do you ever lose consciousness?
10. Do you have a bone or joint problem that could be made worse by performing the movement of standing up from a chair?
11. Is your doctor currently prescribing drugs for your blood pressure or heart condition?
    a. If YES: Has your doctor confirmed that it is safe for you to be physically active while on your blood pressure medication?
12. Do you know of any other reason why you should not do physical activity?

Doc. Suppl. 1. Sit2stand.ai Website Content

Instructions and Upload Page

[See page here]

Instruction Video

Please watch the instruction video and read the instructions fully before performing the test.
[Video]

Sit-to-Stand Instructions

Set-Up

- Go to a well-lit room
- Find a chair that is:
    - Sturdy
    - Free-standing (not against a wall)
    - Without wheels
    - Not a couch or reclining chair
- Place the chair on a steady surface

Participant Start Position

- Sit comfortably in the chair
- Cross your arms in front of you
- Place your feet flat on the floor

Recorder Position

- 45-degree angle so you can see both the side and front of the participant
- 5-10 feet away so the participant's head and feet stay in the video frame even while they are standing

Start the recording

Sit-To-Stand Test

AS QUICKLY AS YOU CAN 5 TIMES[*]:

- **Stand up straight and sit back down**
    - No stopping between reps

    - Stand up fully
    - Do not touch the back of the chair between reps
    - Do not push off of the chair with the back of your legs
- Have the recorder count each stand aloud
- Finish seated in the chair

*Note: If you experience any unusual discomfort or pain, you should stop the test immediately.

Stop the recording

Finish and Upload

- Upload the video
- Continue to the survey

Record and upload your sit-to-stand test video

[Upload form]

Doc. Suppl. 1. Sit2stand.ai Website Content

Example Assessment Results

[See page here]

Your video with motion analysis

[Original video and OpenPose video side by side with titles "Original Video" and "Motion Analysis"]

Your movement analysis

The angles of your joints

These curves show the angle of your left knee when you perform the sit-to-stand test. One curve corresponds to one repetition. The more similar the curves, the more consistent your movement.

RETURN TO THE MAIN PAGE

Doc. Suppl. 1. Sit2stand.ai Website Content

Our Research

[See page here]

Our Vision

Develop at-home clinical assessments for physical and mental health in order to test and develop accessible health-improving interventions.

Team

[photo]
Melissa Boswell, MS
Ph.D. Candidate and Study Coordinator

[photo]
Lukasz Kidzinski, PhD
Researcher and Data Scientist

[photo]
Jennifer Hicks, PhD
Senior Research Engineer

[photo]
Scott Delp, PhD
Principal Investigator

Learn more about the Neuromuscular Biomechanics Lab at Stanford

Research

[video]

Łukasz Kidziński*, Bryan Yang*, Jennifer Hicks, Apoorva Rajagopal, Scott Delp, Michael Schwartz, "Deep neural networks enable quantitative movement analysis using single-camera videos", Nature Communications (2020)
* These authors contributed equally to this work

Melissa A. Boswell*, Scott D. Uhlrich*, Łukasz Kidziński, Kevin Thomas, Julie A. Kolesar, Garry E. Gold, Gary S. Beaupre, Scott L. Delp, "A neural network to predict

the knee adduction moment in patients with osteoarthritis using anatomical landmarks obtainable from 2D video analysis", Osteoarthritis and Cartilage (2021)
* These authors contributed equally to this work

RETURN TO THE MAIN PAGE

Doc. Suppl. 2. Full participant survey.

Demographics

What is your age in years? Please enter the number only.

What is your height?

What is your weight in pounds?

What sex were you assigned at birth, on your original birth certificate?

- Male
- Female

What is your gender identity? Gender identity is one's innermost concept of self as male, female, a blend of both or neither – how individuals perceive themselves and what they call themselves. One's gender identity can be the same or different from their sex assigned at birth.

- ○ Male
- ○ Female
- ○ Non-Binary
- ○ Other
- ○ Prefer Not to Answer

What is your ethnicity? Check all that apply.

- ☐ White
- ☐ Black or African American
- ☐ Hispanic or Latino or Spanish Origin of any race
- ☐ Asian
- ☐ American Indian or Alaskan Native
- ☐ Native Hawaiian or Pacific Islander
- ☐ [ ] Other
- ☐ Prefer Not to Answer

In which country were you **born**?

In which US state do you **currently reside**?

What is the highest degree or level of school you have completed?

- Less than high school
- High school graduate
- Some college
- College Degree or Higher
- Prefer Not to Answer

What is your current employment status?

- Employed full time (40+ hours per week)
- Employed part time (less than 40 hours per week)
- Unemployed and currently looking for work
- Unemployed and not currently looking for work

- Student
- Retired
- Homemaker
- Self-employed
- Unable to work
- Prefer Not to Answer

What is your total household income?

- < $10,000
- $10,000 to $24,999
- $25,000 to $49,999
- $50,000 to $99,999
- > $100,000
- Prefer Not to Answer

What is your current marital status?

- Married
- Widowed
- Divorced
- Separated
- Never married
- Member of an unmarried couple
- Prefer Not to Answer

Are you a current smoker?

- ○ Yes
- ○ No

In the average week, how often do you practice meditation, mindfulness, or prayer meditation?

- ○ Not at all
- ○ 0 - 2 hours
- ○ 2 - 5 hours
- ○ 5 - 10 hours
- ○ More than 10 hours

Do you have any of the following medical conditions (select all that apply)?

- ☐ Heart disease
- ☐ High blood pressure
- ☐ Lung disease
- ☐ Diabetes
- ☐ Ulcer or stomach disease
- ☐ Kidney disease

- ☐ Liver disease
- ☐ Anemia or other blood disease
- ☐ Cancer
- ☐ Depression
- ☐ Osteoarthritis, degenerative arthritis
- ☐ Back pain
- ☐ Rheumatoid arthritis
- ☐ Total joint replacement in the ankle(s), knee(s), or hip(s), please specify:
- ☐ Orthopedic surgery within the past year, please specify:
- ☐ Other medical condition
- ☐ None of the above

If you checked any of the above medical conditions, does it limit your physical activity participation?

- ○ Yes
- ○ No
- ○ N/A

Global Health: PROMIS Scale v1.2 – Global Health

Please respond to each question or statement by marking one box per row.

	Excellent	Very Good	Good	Fair	Poor
In general, would you say your health is:	○	○	○	○	○
In general, would you say your quality of life is:	○	○	○	○	○
In general, how would you rate your physical health?	○	○	○	○	○
In general, how would you rate your mental health, including your mood and your ability to think?	○	○	○	○	○
In general, how would you rate your satisfaction with your social activities and relationships?	○	○	○	○	○
In general, please rate how well you carry out your usual social activities and roles. (This includes activities at home, at work and in your community, and responsibilities as a parent, child, spouse, employee, friend, etc.)	○	○	○	○	○

To what extent are you able to carry out your everyday physical activities such as walking, climbing stairs, carrying groceries, or moving a chair?

- Completely
- Mostly
- Moderately
- A little
- Not at all

How often have you been bothered by emotional problems such as feeling anxious, depressed or irritable over the past 7 days?

- Never
- Rarely
- Sometimes
- Often
- Always

How would you rate your fatigue on average over the past 7 days?

- None
- Mild

- Moderate
- Severe
- Very Severe

How would you rate your pain on average over the past 7 days?

- 0 (No Pain)
- 1
- 2
- 3
- 4
- 5
- 6
- 7
- 8
- 9
- 10 (Worst Pain Imaginable)

Osteoarthritis Questions

Do you have a clinical diagnosis of knee osteoarthritis in at least one knee or hip (i.e. have had a doctor's diagnosis of knee or hip osteoarthritis)?

- ◯ Yes
- ◯ No

Please select the location(s) of your osteoarthritis:

- ☐ Right knee
- ☐ Left knee
- ☐ Right hip
- ☐ Left hip

Have you had a Total Knee or Hip Arthroplasty surgery or other knee or hip surgery after your diagnosis of osteoarthritis?

- ◯ Yes
- ◯ No

Do you have a knee/hip replacement surgery scheduled for sometime in the next 6 months?

- ◯ Yes
- ◯ No

How many years has it been since you were first clinically diagnosed with knee osteoarthritis?

The following questions concern the amount of difficulty you have experienced due to osteoarthritis in your knee(s)/hip(s).

Rate your difficulty when performing the following activities...

	None	Slight	Moderate	Severe	Extreme
Descending stairs	○	○	○	○	○
Ascending stairs	○	○	○	○	○
Rising from sitting	○	○	○	○	○
Standing	○	○	○	○	○
Bending to floor	○	○	○	○	○
Walking on even floor	○	○	○	○	○
Getting in/out of car	○	○	○	○	○
Going shopping	○	○	○	○	○
Putting on socks	○	○	○	○	○

	None	Slight	Moderate	Severe	Extreme
Rising from bed	○	○	○	○	○
Taking off socks	○	○	○	○	○
Lying in bed	○	○	○	○	○
Getting in/out of bath	○	○	○	○	○
Sitting	○	○	○	○	○
Getting on/off toilet	○	○	○	○	○
Doing light domestic duties (cooking, dusting)	○	○	○	○	○

The following questions concern the amount of pain you have experienced due to osteoarthritis in your knee(s)/hip(s).

Rate your pain in your knee(s)/hips(s) when...

	None	Slight	Moderate	Severe	Extreme
Walking	○	○	○	○	○
Climbing Stairs	○	○	○	○	○
Sleeping at night	○	○	○	○	○
Resting	○	○	○	○	○
Standing	○	○	○	○	○

The following questions concern the amount of stiffness you have experienced due to osteoarthritis in your knee(s)/hip(s).

Rate your stiffness in your knee(s)/hip(s) in the...

	None	Slight	Moderate	Severe	Extreme
Morning	○	○	○	○	○
Evening	○	○	○	○	○

Mood

Please choose one (or more) expressions that best describe your current mood:

- ☐ Excited / Lively
- ☐ Cheerful / Happy
- ☐ Calm / Serene
- ☐ Relaxed / Carefree
- ☐ Bored / Weary
- ☐ Gloomy / Sad
- ☐ Irritated / Annoyed
- ☐ Tense / Nervous

Falls Survey (Adapted from Mertz, 2010)

Have you fallen in the past 12 months?

- Yes
- No

Have you had multiple falls in the past 12 months?

- Yes
- No

Approximately how many falls have you had in the past 12 months? Please enter a number.

[ ]

If you have had a fall in the past 12 months, what was the activity of the most recent fall?

- ○ Sports/exercise
- ○ Walking
- ○ On a stool or ladder
- ○ Getting up from a lying or sitting position
- ○ Getting in or out of the tub or shower
- ○ [ ] Other

Did you fracture any bones due to the most recent fall?

- ○ Yes
- ○ No

Physical activity tracker

Do you use an app on your phone or a wearable device (i.e., watch or pedometer) that tracks your **daily step count**?

- ○ Yes
- ○ No

For the next few questions, please refer to your activity tracker app or log. **Note: Do not close this window entirely.** You can leave it up while you open your activity tracker app.

Would you say that past 5 days are representative of your usual physical activity level?

- ○ Yes
- ○ No, I was more active than usual
- ○ No, I was less active than usual

What is your target step count currently set at?

[ ]

Please enter your total **daily step count** over the past 5 days, starting with yesterday.

1 day ago (yesterday)

2 days ago

3 days ago

4 days ago

5 days ago

Do you use an app on your phone or a wearable device (i.e., watch or pedometer) that tracks your **daily active time**?

- Yes
- No

What is your target daily active time set at?

Please enter your total **daily active time in minutes** for the past 5 days, starting with yesterday.

1 day ago (yesterday) in minutes

2 days ago

3 days ago

4 days ago

5 days ago

International Physical Activity Questionnaire - Short Form

We are interested in finding out about the kinds of physical activities that people do as part of their everyday lives. The questions will ask you about the time you spent being physically active in the last 7 days. Please answer each question even if you do not consider yourself to be an active person. Please think about the activities you do at work, as part of your house and yard work, to get from place to place, and in your spare time for recreation, exercise or sport.

Think about all the vigorous activities that you did in the last 7 days. Vigorous physical activities refer to activities that take hard physical effort and make you breathe much harder than normal. Think only about those physical activities that you did for at least 10 minutes at a time.

During the last 7 days, on how many days did you do vigorous physical activities like heavy lifting, digging, aerobics, or fast bicycling?

- ○ [ ] Days per week
- ○ No vigorous physical activities

How much time did you usually spend doing vigorous physical activities on one of those days?

Minutes per day

Think about all the moderate activities that you did in the last 7 days. Moderate activities refer to activities that take moderate physical effort and make you breathe somewhat harder than normal. Think only about those physical activities that you did for at least 10 minutes at a time.

During the last 7 days, on how many days did you do moderate physical activities like carrying light loads, bicycling at a regular pace, or doubles tennis? **Do not include walking.**

- ○ Days per week
- ○ No moderate physical activities

How much time did you usually spend doing moderate physical activities on one of those days?

Minutes per day

Think about the time you spent walking in the last 7 days. This includes at work and at home, walking to travel from place to place, and any other walking that you have done solely for recreation, sport, exercise, or leisure.

During the last 7 days, on how many days did you walk for at least 10 minutes at a time?

- ○ ______ Days per week
- ○ No walking

How much time did you usually spend walking on one of those days?

Hours per day

This question is about the time you spent sitting on weekdays during the last 7 days. Include time spent at work, at home, while doing course work and during leisure time. This may include time spent sitting at a desk, visiting friends, reading, or sitting or lying down to watch television.

During the last 7 days, how much time did you spend sitting on a week day?

Hours per day ☐

Process of Exercise Scale

The following statements are different opinions about what it is like to exercise. Please select the option on each row that best describes how you feel about the process of **exercising**. There are no correct answers – we are only interested in your personal beliefs.

Exercising is:

○ Very difficult

- Somewhat difficult
- Somewhat easy
- Very easy

Exercising is:

- Very unpleasant
- Somewhat unpleasant
- Somewhat pleasurable
- Very pleasurable

Exercising is:

- Very stressful
- Somewhat stressful
- Somewhat relaxing
- Very relaxing

Exercising is:

- Very depriving
- Somewhat depriving
- Somewhat indulgent
- Very indulgent

Exercising is:

- Very boring
- Somewhat boring
- Somewhat fun
- Very fun

Exercising is:

- Very lonely
- Somewhat lonely
- Somewhat social
- Very social

Exercising is:

- Very inconvenient
- Somewhat inconvenient
- Somewhat convenient
- Very convenient

Exercise Benefits Mindset Measure (Adequacy)

In answering the next few questions, please **think about your current level of physical activity or inactivity** (i.e., how much exercise, if any, you have been doing recently).

How much do you agree or disagree with the following statement?

My current level of physical activity is **unhealthy**.

- Strongly disagree
- Disagree
- Somewhat disagree
- Neither agree nor disagree
- Somewhat agree
- Agree
- Strongly agree

How much do you agree or disagree with the following statement?

My current level of physical activity is helping me **achieve or maintain a healthy body weight**.

- Strongly disagree
- Disagree
- Somewhat disagree
- Neither agree nor disagree
- Somewhat agree
- Agree
- Strongly agree

How **beneficial** is your current level of physical activity for your health?

- Extremely beneficial
- Very beneficial
- Moderately beneficial
- Slightly beneficial
- Not at all beneficial

How much does your current level of physical activity or physical inactivity **increase or decrease your risk of disease**?

- Increases my risk very much

- Increases my risk moderately
- Increases my risk slightly
- Neither increases nor decreases my risk
- Decreases my risk slightly
- Decreases my risk moderately
- Decreases my risk very much

How much is your current level of physical activity or physical inactivity **strengthening or weakening your muscles?**

- Strengthening very much
- Strengthening moderately
- Strengthening slightly
- Neither strengthening nor weakening
- Weakening slightly
- Weakening moderately
- Weakening very much

Falls Efficacy Scale - International - Short Form

Now we would like to ask some questions about how concerned you are about the possibility of falling. Please

reply thinking about how you usually do the activity. If you currently don’t do the activity, please answer to show whether you think you would be concerned about falling IF you did the activity.

For each of the following activities, please tick the box which is closest to your own opinion to show how concerned you are that you might fall if you did this activity

	Not concerned at all	Somewhat concerned	Fairly concerned	Very concerned
Getting dressed or undressed	○	○	○	○
Taking a bath or shower	○	○	○	○
Getting in or out of a chair	○	○	○	○
Going up or down stairs	○	○	○	○
Reaching for something above your head or on the ground	○	○	○	○
Walking up or down a slope	○	○	○	○
Going out to a social event (e.g. religious service, family gathering or club meeting)	○	○	○	○

Self-Efficacy for Exercise Scale (SEE)

How confident are you right now that you could exercise three times per week for 20 minutes if:

	0 (Not Confident)	1	2	3	4	5	6	7	8	9	10 (Very Confident)
The weather was bothering you	○	○	○	○	○	○	○	○	○	○	○
You were bored by the program or activity	○	○	○	○	○	○	○	○	○	○	○
You felt pain when exercising	○	○	○	○	○	○	○	○	○	○	○
You had to exercise alone	○	○	○	○	○	○	○	○	○	○	○
You did not enjoy it	○	○	○	○	○	○	○	○	○	○	○
You were too busy with other activities	○	○	○	○	○	○	○	○	○	○	○
You felt tired	○	○	○	○	○	○	○	○	○	○	○
You felt stressed	○	○	○	○	○	○	○	○	○	○	○
You felt depressed	○	○	○	○	○	○	○	○	○	○	○

Feedback

Thank you for your participation so far! Before we direct you to the end of the survey, we would greatly appreciate your feedback on what it was like to participate in this study. Your response will help us improve this tool for future use.

How easy or difficult was it for you to complete the sit-to-stand test portion of this study (including reading the instructions, performing the test, and uploading the video)?

Very difficult	Somewhat difficult	Neither difficult nor easy	Somewhat easy	Very easy
○	○	○	○	○

Was anything confusing or difficult about the sit-to-stand test portion of this study (including reading the instructions, performing the test, and uploading the video)?

Is there any additional information you would like to share about your experience with this study in general?

Follow-Up

If you would be willing to participate in a follow-up survey or study, please enter your e-mail address:

Continue

For an second entry into the lottery for a $100 Amazon gift card, you can answer an additional set of questions (approximately 10 more minutes). Would you like to continue to additional questions?

- Yes, I'd like an extra lottery entry!
- No

Illness Mindset Inventory

Below are some statements about the body and knee osteoarthritis. Choose the option that best reflects how much you generally agree or disagree with each statement. There are no correct answers – we are only interested in your personal beliefs.

	Strongly Disagree	Disagree	Somewhat Disagree	Somewhat Agree	Agree	Strongly Agree
Your body can heal itself on its own in many different circumstances.	○	○	○	○	○	○
Chronic illness negatively impacts nearly all parts of life.	○	○	○	○	○	○
Having a chronic illness means that your body isn't doing its job.	○	○	○	○	○	○
Your body is able to cope with a chronic illness.	○	○	○	○	○	○
A chronic illness can be an opportunity to make positive life changes	○	○	○	○	○	○
A chronic illness is manageable.	○	○	○	○	○	○
If you have a chronic illness, it means your body has betrayed you.	○	○	○	○	○	○

	Strongly Disagree	Disagree	Somewhat Disagree	Somewhat Agree	Agree	Strongly Agree
	Strongly Disagree	Disagree	Somewhat Disagree	Somewhat Agree	Agree	Strongly Agree
Having a chronic illness allows you to find more meaning in life.	◯	◯	◯	◯	◯	◯
A chronic illness is something that can be dealt with.	◯	◯	◯	◯	◯	◯
In general, your body has remarkable self-healing properties.	◯	◯	◯	◯	◯	◯
Having a chronic illness spoils many parts of life.	◯	◯	◯	◯	◯	◯
Having a chronic illness is a challenge that can make you stronger.	◯	◯	◯	◯	◯	◯
In general, your body is able to handle a chronic illness.	◯	◯	◯	◯	◯	◯
A chronic illness ruins most aspects of life.	◯	◯	◯	◯	◯	◯
	Strongly Disagree	Disagree	Somewhat Disagree	Somewhat Agree	Agree	Strongly Agree
Your body is designed to deal with and manage a chronic illness.	◯	◯	◯	◯	◯	◯

	Strongly Disagree	Disagree	Somewhat Disagree	Somewhat Agree	Agree	Strongly Agree
You can live a relatively normal life with a chronic illness.	○	○	○	○	○	○
Having a chronic illness means that your body has failed.	○	○	○	○	○	○
Fighting a chronic illness can be empowering.	○	○	○	○	○	○
Your body is able to heal itself from most conditions and diseases.	○	○	○	○	○	○
Your body is to blame if you have a chronic illness.	○	○	○	○	○	○

Physical Sensations as Signals

When you experience physical sensations during exercise (for example, sweating, being out of breath, or muscle fatigue), how discouraged do you feel?

Very discouraged	Kind of discouraged	Not that discouraged	Not discouraged at all
○	○	○	○

When you experience physical sensations during exercise (for example, sweating, being out of breath, or muscle fatigue), how do you think the exercise is going?

Very poorly	Poorly	Well	Very well
○	○	○	○

When you experience physical sensations during exercise (for example, sweating, being out of breath, or muscle fatigue), do you think your body is getting stronger?

Not at all	Not really	Kind of	Definitely
○	○	○	○

When you experience physical sensations during exercise (for example, sweating, being out of breath, or muscle fatigue), do you think the exercise is working for you?

Not at all	Not really	Kind of	Definitely
○	○	○	○

When you experience physical sensations during exercise (for example, sweating, being out of breath, or muscle fatigue), do you think your body is weak or out of shape?

Not at all	Not really	Kind of	Definitely
○	○	○	○

When you experience physical sensations during exercise (for example, sweating, being out of breath, or muscle fatigue), do you think the exercise is **not** working for you?

Not at all	Not really	Kind of	Definitely
○	○	○	○

hen you experience physical sensations during exercise (for example, sweating, being out of breath, or muscle fatigue), how likely are you to persist and continue the exercise?

Not at all	Not really	Kind of	Definitely
○	○	○	○

Fatigue

Please circle the number between 1 and 7 which you feel best fits the following statements. This refers to your usual way of life within the last week. 1 indicates “strongly disagree” and 7 indicates “strongly agree.”

Please read and choose a response.

	Strongly Disagree	Disagree	Somewhat Disagree	Neither Agree Nor Disagree	Somewhat Agree	Agree	Strongly Agree
My motivation is lower when I am fatigued.	○	○	○	○	○	○	○

	Strongly Disagree	Disagree	Somewhat Disagree	Neither Agree Nor Disagree	Somewhat Agree	Agree	Strongly Agree
Exercise brings on my fatigue.	○	○	○	○	○	○	○
I am easily fatigued.	○	○	○	○	○	○	○
Fatigue interferes with my physical functioning.	○	○	○	○	○	○	○
Fatigue causes frequent problems for me.	○	○	○	○	○	○	○
My fatigue prevents sustained physical functioning.	○	○	○	○	○	○	○
Fatigue interferes with carrying out certain duties and responsibilities.	○	○	○	○	○	○	○
Fatigue is among my most disabling symptoms.	○	○	○	○	○	○	○
Fatigue interferes with my work, family, or social life.	○	○	○	○	○	○	○

Powered by Qualtrics
