# Supplementary material for: Smartphone videos of the sit-to-stand test predict osteoarthritis and health outcomes in a nationwide study
Source: NPJ Digit Med. 2023 Mar 4;6:32. doi: 10.1038/s41746-023-00775-1 (PMC9985590; doi:10.1038/s41746-023-00775-1)
Supplement: Supplementary file 2 — REPORTING SUMMARY [file 41746_2023_775_MOESM2_ESM.pdf]

nature portfolio

Corresponding author(s): Melissa Boswell, Łukasz Kidziński, Jennifer Hicks, Scott Uhlrich, Antoine Falisse, Scott Delp

Last updated by author(s): Dec 21, 2022

Reporting Summary

Nature Portfolio wishes to improve the reproducibility of the work that we publish. This form provides structure for consistency and transparency in reporting. For further information on Nature Portfolio policies, see our Editorial Policies and the Editorial Policy Checklist.

Statistics

For all statistical analyses, confirm that the following items are present in the figure legend, table legend, main text, or Methods section.

n/a	Confirmed	
☐	☒	The exact sample size (*n*) for each experimental group/condition, given as a discrete number and unit of measurement
☐	☒	A statement on whether measurements were taken from distinct samples or whether the same sample was measured repeatedly
☐	☒	The statistical test(s) used AND whether they are one- or two-sided<br>*Only common tests should be described solely by name; describe more complex techniques in the Methods section.*
☐	☒	A description of all covariates tested
☐	☒	A description of any assumptions or corrections, such as tests of normality and adjustment for multiple comparisons
☐	☒	A full description of the statistical parameters including central tendency (e.g. means) or other basic estimates (e.g. regression coefficient) AND variation (e.g. standard deviation) or associated estimates of uncertainty (e.g. confidence intervals)
☐	☒	For null hypothesis testing, the test statistic (e.g. *F*, *t*, *r*) with confidence intervals, effect sizes, degrees of freedom and *P* value noted<br>*Give P values as exact values whenever suitable.*
☒	☐	For Bayesian analysis, information on the choice of priors and Markov chain Monte Carlo settings
☒	☐	For hierarchical and complex designs, identification of the appropriate level for tests and full reporting of outcomes
☐	☒	Estimates of effect sizes (e.g. Cohen's *d*, Pearson's *r*), indicating how they were calculated

Our web collection on statistics for biologists contains articles on many of the points above.

Software and code

Policy information about availability of computer code

Data collection	We processed all videos using OpenPose, an open-source neural network-based software for pose-estimation. Custom scripts used for data collection are open source at github.com/stanfordnmbl/motionlab-analysis.
Data analysis	R version 4.2.1 was used with the base packages and the following additional packages: stddiff, tidyverse, jtools, FSA, ggpubr, readxl, psych, and sjmisc. Custom scripts used for data processing and analysis and are open source at github.com/stanfordnmbl/motionlab-analysis.

For manuscripts utilizing custom algorithms or software that are central to the research but not yet described in published literature, software must be made available to editors and reviewers. We strongly encourage code deposition in a community repository (e.g. GitHub). See the Nature Portfolio guidelines for submitting code & software for further information.

Data

Policy information about availability of data

All manuscripts must include a data availability statement. This statement should provide the following information, where applicable:

- Accession codes, unique identifiers, or web links for publicly available datasets
- A description of any restrictions on data availability
- For clinical datasets or third party data, please ensure that the statement adheres to our policy

All data generated in this study have been de-identified and are available on GitHub: github.com/stanfordnmbl/motionlab-analysis. A small subset of three videos

obtained had identifiers that could not be sufficiently removed and, thus, will not be available publicly. However, the processed results of these videos are still included in the available dataset.

Human research participants

Policy information about studies involving human research participants and Sex and Gender in Research.

Reporting on sex and gender	Sex and gender were both collected in this study through a self-report survey. These measures are reported disaggregated in participant characteristics. Participants consented to share de-identified data, including reporting of sex and gender. Differences in outcome measures were analyzed across sex. Sex was also used as an independent variable in regression analyses. We did not recruit for a specific ratio of sexes and obtained a 45/55 ratio of female to male identification.
Population characteristics	Age, sex, and BMI were used as control variables in some regression analyses. We also assessed physical health, mental health, ethnicity and race, and diagnosis of knee/hip osteoarthritis. These characteristics, and other participant characteristics collected, are detailed in our manuscript.
Recruitment	Participants were recruited participants via social media postings, flyers, word of mouth, and other studies' participant pools. By leveraging research studies focusing on aging and osteoarthritis, we recruited individuals of older age and with hip and/or knee osteoarthritis.
Ethics oversight	Stanford University Institutional Review Board

Note that full information on the approval of the study protocol must also be provided in the manuscript.

Field-specific reporting

Please select the one below that is the best fit for your research. If you are not sure, read the appropriate sections before making your selection.

☒ Life sciences ☐ Behavioural & social sciences ☐ Ecological, evolutionary & environmental sciences

For a reference copy of the document with all sections, see nature.com/documents/nr-reporting-summary-flat.pdf

Life sciences study design

All studies must disclose on these points even when the disclosure is negative.

Sample size	No sample-size was chosen. We aimed to collect a diverse nation-wide sample to perform exploratory analyses.
Data exclusions	Out of 493 videos submitted, 489 were successfully processed with OpenPose. From this subset, we excluded 84 participants due to the following video recording errors (not mutually exclusive): use of a heavily cushioned chair (n=2); long pause between repetitions (n=29); too close, out of frame, or bodily obstruction (n=25); camera angle was planar (rather than at a 45-degree angle; n=34); use of arms to stand (n=20); large pose-estimation error due to participant wearing a skirt (n=1). This criteria was not pre-established.
Replication	Our web app, data set, source code, and processing code are freely available online, enabling replication.
Randomization	Randomization is not relevant to our study as we performed a cross-sectional evaluation.
Blinding	Blinding was not relevant to our study as we did not have multiple participant groups.

Reporting for specific materials, systems and methods

We require information from authors about some types of materials, experimental systems and methods used in many studies. Here, indicate whether each material, system or method listed is relevant to your study. If you are not sure if a list item applies to your research, read the appropriate section before selecting a response.

Materials & experimental systems

n/a	Involved in the study	
☒	☐	Antibodies
☒	☐	Eukaryotic cell lines
☒	☐	Palaeontology and archaeology
☒	☐	Animals and other organisms
☒	☐	Clinical data
☒	☐	Dual use research of concern

Methods

n/a	Involved in the study	
☒	☐	ChIP-seq
☒	☐	Flow cytometry
☒	☐	MRI-based neuroimaging
